# Association of rs9939609-FTO with metabolic syndrome components among women from Mayan communities of Chiapas, Mexico

**DOI:** 10.1186/s40101-021-00259-9

**Published:** 2021-08-28

**Authors:** Pilar E. Núñez Ortega, María E. Meneses, Iván Delgado-Enciso, César Antonio Irecta-Nájera, Itandehui Castro-Quezada, Roberto Solís-Hernández, Elena Flores-Guillén, Rosario García-Miranda, Adán Valladares-Salgado, Daniel Locia-Morales, Héctor Ochoa-Díaz-López

**Affiliations:** 1 Health Department, El Colegio de La Frontera Sur, San Cristóbal de Las Casas, Chiapas, Mexico; 2 National Council for Science and Technology, Postgraduate College Campus Puebla, Puebla, Mexico; 3 Facultad de Medicina, Universidad de Colima, Colima, Mexico; 4 Instituto Estatal de Cancer, Secretaria de Salud de Colima, Colima, Mexico; 5 Health Department, El Colegio de La Frontera Sur, Villahermosa, Tabasco Mexico; 6 Faculty of Nutrition and Food Science, University of Science and Arts of Chiapas, Tuxtla Gutiérrez, Chiapas Mexico; 7 School of Languages-Campus San Cristobal, Autonomous University of Chiapas, San Cristóbal de Las Casas, Chiapas Mexico; 8 Unidad de Investigación Médica en Bioquímica, Hospital de Especialidades, Centro Médico Nacional Siglo XXI. Instituto Mexicano del Seguro Social, Mexico City, Mexico

**Keywords:** Metabolic syndrome, Single nucleotide polymorphisms, FTO, Mayan indigenous women, Chiapas, Mexico

## Abstract

**Background:**

Metabolic syndrome (MetS) is a complex cluster of risk factors, considered as a polygenic and multifactorial entity. The objective of this study was to determine the association of rs9939609-FTO polymorphism and MetS components in adult women of Mayan communities of Chiapas.

**Methods:**

In a cross-sectional study, sociodemographic, anthropometric, clinical, and biochemical data were obtained from 291 adult women from three regions of Chiapas, Mexico. The prevalence of MetS and the allele and genotype frequencies of the rs9939609-FTO were estimated. Multivariate logistic regression models were used to assess the association of the single nucleotide polymorphism (SNP) with each of the MetS components.

**Results:**

The MetS prevalence was 60%. We found a statistically significant association between rs9939609-FTO and hyperglycemia in the dominant model (OR 2.6; 95% CI 1.3–5.3; *p* = 0.007).

**Conclusions:**

Women from Mayan communities of Chiapas presented a high prevalence of MetS and a relevant association of the FTO variant with hyperglycemia. This is the first study carried out in these Mayan indigenous communities from Chiapas.

## Background

Chronic diseases are one of the biggest challenges that Mexico’s health system is facing [1]. This is due to their high prevalence, great contribution to overall mortality, premature disability, and high costs of their treatment. Metabolic syndrome (MetS) is characterized by the presence of insulin resistance, hyperglycemia and/or type 2 diabetes (T2D), dyslipidemias, abdominal obesity, high blood pressure (HBP), and endothelial dysfunction [2]. All these alterations may sequentially or simultaneously be present in MetS, potentially contribute to the development of cardiovascular diseases (CVD) [3], and confer a high risk of morbidity/mortality [4].

Due to the complexity of this set of pathologies, comprehensive studies are currently carried out for a better understanding of its pathophysiology. MetS has been considered a polygenic and multifactorial entity [5]. Family and population studies show that MetS is influenced by a strong genetic component, with great variability among different ethnic groups. In fact, 45% of first-grade family members of patients with T2D, even at normal glucose levels, show to have insulin resistance [6].

In our study, we evaluated the single nucleotide polymorphism (SNP), rs9939609, located at the first intron of the FTO gene. This SNP is one of the most extensively studied, explaining approximately 1% of body mass index (BMI) heritability [7]. In addition, several studies have systematically confirmed the association of a group of SNPs in the first intron of this gene with obesity-related traits in Europeans [7–9], Asian [10, 11], and African populations [12, 13].

With regard to the Mexican mestizo population, there are studies that have shown the association of this genetic variant with the development of the pathologies involved in MetS [14, 15]. Although the function of the FTO protein has not been clearly elucidated, some previous studies linked this protein to impaired fasting glucose and insulin resistance [16–18]. However, the association of this FTO variant has not been studied in Mayan indigenous communities from Chiapas, Mexico.

The objective of this research was to determine the prevalence of MetS and the allele frequency of the SNP rs9939609-FTO as well as its association with the components of Mets in women from Mayan indigenous communities of Chiapas, Mexico.

## Methods

### Study population

**Fig. 1:**
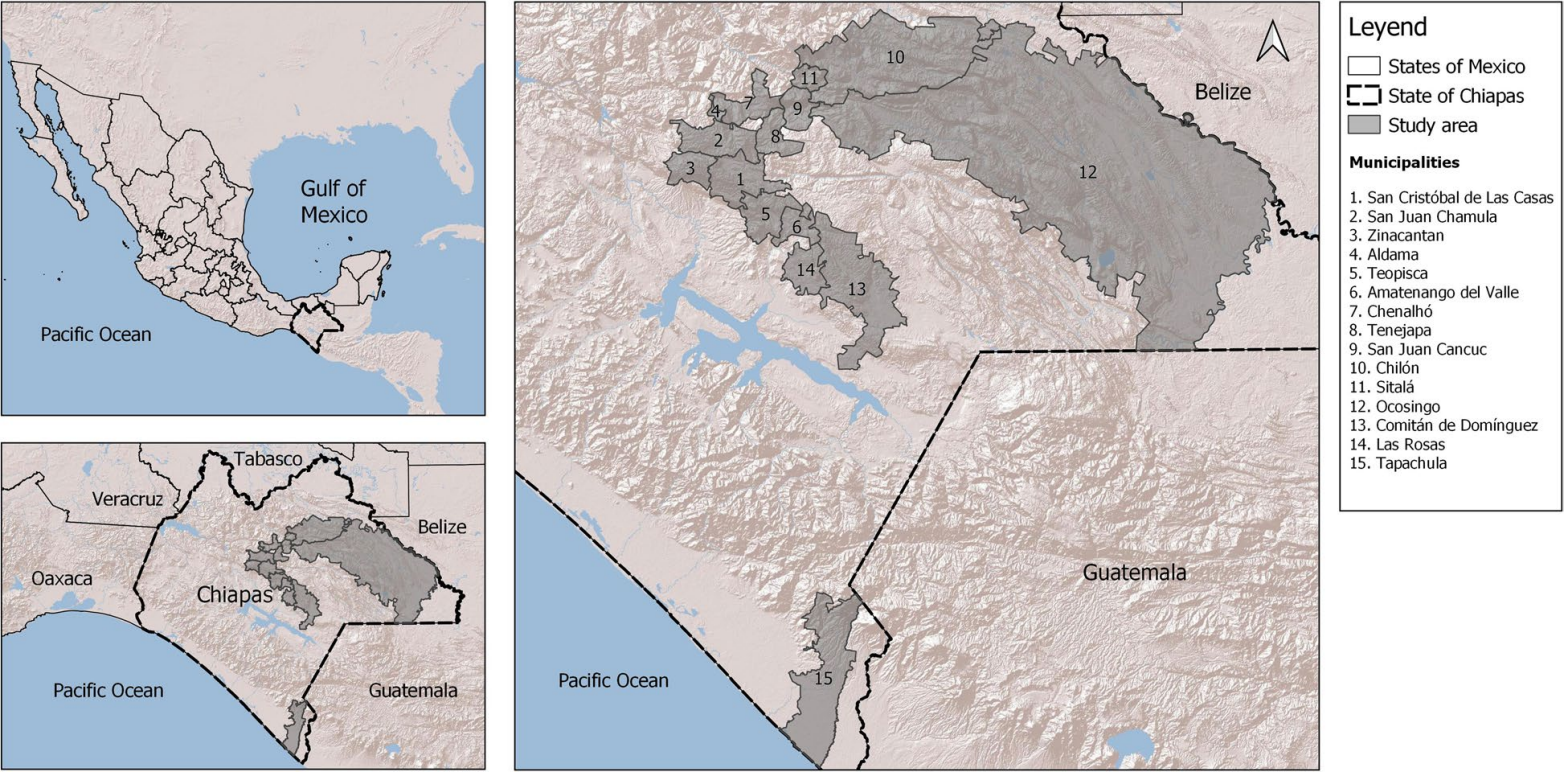
Study area: Tzotzil-Tzeltal, Selva, and Soconusco regions of Chiapas, Mexico

The study population belongs to three regions of Mayan ancestry of Chiapas, Mexico: Tzotzil-Tzeltal (11 communities), Selva (79 communities), and Soconusco (2 communities). Data were collected from two cross-sectional studies conducted in 2017–2018 in the regions mentioned above [19, 20]. In total, 310 women participated in these studies. A high percentage of the general population of these regions belongs to marginalized and extremely poor indigenous groups (Fig. 1). Participants with missing information in the main variables of this study were excluded (*n* = 12) from the analysis. The final sample included 291 individuals. All participants gave their informed consent for inclusion in the study.

### Data collection

A validated structured questionnaire was applied with the following sections: sociodemographic data, non-pathological personal history, family medical history, anthropometric and clinical measures, and frequency of food consumption. Sociodemographic data included age, geographic area, ethnicity and years of schooling, household items, and type of cooking fuel.

Family medical history included first and second degree of consanguinity relatives’ diseases: obesity, diabetes, HBP, and CVD. Non-pathological personal history included smoking and alcohol consumption.

### Anthropometric and clinical assessment

Weight (kg) was measured by electronic scales (Model UM081, Tanita Corporation, accuracy ± 100 g, Tokyo, Japan). Height (m) was measured using stadiometers (SECA, accuracy ± 1 mm, Berlin, Germany). Waist circumference was measured by anthropometric tapes (SECA, precision ± 1 mm, Berlin, Germany). BMI was estimated as weight divided by height squared. Then, weight status was categorized as follows: underweight and normal weight (BMI < 25 kg/m^2^) and overweight or obesity (BMI ≥ 25 kg/m^2^).

Blood pressure was measured twice, using a digital monitor (Model CH-453, Citizen, Japan). The readings were taken with the participant seated and after a 5-min rest.

### Frequency of food consumption

The frequency of food intake was assessed by using a 37-item food frequency questionnaire (FFQ) [19]. Participants were asked on how often they consumed each food over 1 week. The frequency of food intake was categorized as follows: 0–1, 2–4, or 5 or more times per week.

### Biochemical measurements

Fasting 5-mL blood samples (10 h) were taken from the antecubital vein for biochemical analysis. The determinations of serum glucose, triglycerides, and HDL-c were performed by photometric enzymatic methods (Diasys, Diagnostic System, Holzheim, Germany), in an automated analyzer (Vitalab Selectra E, Vitalab Scientific, Île-de-France, France).

### Classification of metabolic syndrome (MetS)

MetS was identified using the criteria of the Joint Statement of International Diabetes Federation Task Force on Epidemiology and Prevention; National Heart, Lung, and Blood Institute; American Heart Association; World Heart Federation; International Atherosclerosis Society; and International Association for the Study of Obesity [21]. MetS was defined as the presence of three or more of the following conditions: elevated waist circumference (≥ 80 cm); elevated triglycerides (≥ 150 mg/dL) or drug treatment for elevated triglycerides; reduced HDL-c < 50 mg/dL or drug treatment for reduced HDL-c; systolic blood pressure (SBP) ≥ 130 mm Hg or diastolic blood pressure (DBP) ≥ 85 mmHg or antihypertensive drug treatment in a patient with a history of hypertension; and fasting glucose levels ≥ 100 mg/dL or drug treatment for elevated glucose.

### Genotyping

The isolation of genomic DNA was performed in ECOSUR Health Laboratory at San Cristobal de Las Casas, Chiapas, using commercial kits based on columns (Universal Quick-DNATM Kit/Zymo Research, USA). The DNA samples were frozen at − 42 °C and transported to the Biochemistry Unit of the Centro Médico Nacional Siglo XXI (National Medical Center of the 21st Century) in Mexico City, where all the molecular analyses were performed. The purity and concentration of genomic DNA were verified by spectrophotometry at 260/280 nm (Epoch, Biotek, Winooski, Vermont), and the integrity of the DNA was confirmed by electrophoresis in an agarose gel at 0.8%. The analysis of the SNP rs9939609-FTO was made using TaqMan-probe-based real-time PCR (7900HT Applied Biosystems, Foster City, CA, USA), following standard protocols. A concordance of 100% was observed in 30 duplicate samples for quality control of each probe.

### Statistical analysis

A descriptive analysis of the variables was performed by MetS using percentages and 95% confidence intervals (95% CI) for categorical variables. For continuous variables, we have conducted the Shapiro–Wilk test for normal data distribution. Medians and interquartile ranges were calculated for skewed biochemical and dietary measurements. Differences between groups were analyzed using chi-square tests for categorical variables and Mann- Whitney *U* tests for continuous variables. Allele and genotype frequencies were estimated. Hardy–Weinberg equilibrium (HWE) was estimated for the variant under study. To assess the association between MetS components and rs9939609-FTO, logistic regression models assuming three different modes of inheritance (codominant, dominant, and recessive) were fitted. Odds ratios (ORs) and 95% CI were estimated to measure the magnitude of association between rs9939609-FTO and MetS components. Models were adjusted for age (years, continuous), BMI (kg/m^2^, continuous), schooling (years, continuous), and presence of T2D. For all the analyses, we considered a *p*-value of ≤ 0.05 as a significant level. To assess a possible interaction between geographic region or previously diagnosed hypertension and rs9939609-FTO, we introduced the product terms of the variables in the logistic regression models and considered *p* < 0.05 in the likelihood ratio test as statistically significant. All analyses were performed using STATA software (StataCorp, College Station, TX 77,845, USA; version 16.1, 2019).

## Results

### Sociodemographic characteristics of the study population by MetS

The sociodemographic characteristics of the study population by MetS are shown in Table 1. Women over 45 years had the highest prevalence of MetS (75%).

**Table 1:** Sociodemographic characteristics of the study population by MetS

	**Without MetS**				**With MetS**				**Total**		*P*-value*
	*n*	% or median	95% CI or p25–p75		*n*	% or median	95% CI or p25–p75		*n*		
**Characteristics**											
**Age**											
Years	116	37.0	33.0	43.0	175	40.0	35.0	47.0	291	100	0.001†
< 35 years	39	53.4	42.0	64.5	34	46.6	35.5	58.0	73	100	0.006
35–40 years	35	38.9	29.3	49.2	55	61.1	50.8	70.7	90	100	
41–45 years	24	42.9	30.5	55.9	32	57.1	44.1	69.5	56	100	
> 45 years	18	25.0	16.1	35.8	54	75.0	64.2	83.9	72	100	
Total	116	39.9	34.4	45.6	175	60.1	54.4	65.6	291	100	
**Geographic area**											
Urban	94	40.7	34.5	47.1	137	59.3	52.9	65.5	231	100	0.570
Rural	22	36.7	25.3	49.3	38	63.3	50.7	74.7	60	100	
Total	116	39.9	34.4	45.6	175	60.1	54.4	65.6	291	100	
**Years of schooling**											
Years	116	9.0	4.3	12.0	175	6.0	2.0	9.0	291	100	< 0.001†
0–5 years	34	32.1	23.8	41.4	72	67.9	58.6	76.2	106	100	0.008
6–10 years	52	39.1	31.1	47.6	81	60.9	52.4	68.9	133	100	
> 10 years	30	57.7	44.2	70.4	22	42.3	29.6	55.8	52	100	
Total	116	39.9	34.4	45.6	175	60.1	54.4	65.6	291	100	
**Language (ethnicity)**											
Spanish	76	41.1	34.2	48.3	109	58.9	51.7	65.8	185	100	0.575
Indigenous (any Mayan languages)	40	37.7	28.9	47.2	66	62.3	52.8	71.1	106	100	
Total	116	39.9	34.4	45.6	175	60.1	54.40	65.6	291	100	
**Household conditions**											
**Piped water within the house**											
Yes	98	42.2	36.0	48.7	134	57.8	51.3	64.0	232	100	0.100
No	18	30.5	19.9	43.0	41	69.5	57.0	80.1	59	100	
Total	116	39.9	34.4	45.6	175	60.1	54.4	65.6	291	100	
**Cooking fuel**											
Wood or coal	39	47.0	36.5	57.7	44	53.0	42.3	63.5	83	100	0.272
Gas or electric	32	35.6	26.2	45.8	58	64.4	54.2	73.8	90	100	
Both	45	38.1	29.7	47.1	73	61.9	52.9	70.3	118	100	
Total	116	39.9	34.4	45.6	175	60.1	54.4	65.6	291	100	
**Television**											
No	12	37.5	22.4	54.8	20	62.5	45.2	77.6	32	100	0.772
Yes	104	40.2	34.3	46.2	155	59.8	53.8	65.7	259	100	
Total	116	39.9	34.4	45.6	175	60.1	54.4	65.6	291	100	
**Microwave oven**											
No	83	39.5	33.1	46.2	127	60.5	53.8	66.9	210	100	0.849
Yes	33	40.7	30.5	51.6	48	59.3	48.4	69.5	81	100	
Total	116	39.9	34.4	45.6	175	60.1	54.4	65.6	291	100	
**Cell phone (head of the household)**											
No	32	42.7	31.9	54.0	43	57.3	46.0	68.1	75	100	0.565
Yes	84	38.9	32.6	45.5	132	61.1	54.5	67.4	216	100	
Total	116	39.9	34.4	45.6	175	60.1	54.4	65.6	291	100	
**Fridge**											
No	32	38.1	28.3	48.7	52	61.9	51.3	71.7	84	100	0.695
Yes	84	40.6	34.1	47.4	123	59.4	52.6	65.9	207	100	
Total	116	39.9	34.4	45.6	175	60.1	54.4	65.6	291	100	

^*^Chi-square test for independence. †Mann-Whitney *U* test

Women living in rural areas had a higher prevalence of MetS than those living in urban areas (localities of more than 2500 people) which in Mexico represents more than 70% of its geographical area.

Women with less than 5 years of schooling had a higher prevalence of MetS than those with the highest schooling. Women who speak an indigenous language had a slightly higher prevalence of MetS (62%) than women who only speak Spanish (60%). With regard to household conditions, a great proportion of the study population lacks basic amenities such as piped water inside the house, stove, and fridge. However, no marked differences were observed.

### Anthropometric, clinical, and biochemical parameters of the study population by MetS

Table 2 shows the anthropometric, clinical, and biochemical parameters of the study population by MetS. Ninety-two percent of the total participants had a waist circumference greater than 80 cm. A high percentage of them (88%) presented a BMI ≥ 25 kg/m^2^, 61% had high level of triglycerides (≥ 150 mg/dL), and 70% had low HDL-c (< 50 mg/dL). We identified a low percentage of women with alterations in fasting glucose and blood pressure levels (22% and 32%, respectively).

**Table 2:** Anthropometric, clinical, and biochemical parameters of the study population by MetS

**Variables**	**With MetS (*n*)**	**Median (p25–p75) or % (95% CI)**	**Without MetS (*n*)**	**Median (p25–p75) or % (95% CI)**	**Total**	**Median or % (95% CI)**
**Waist circumference**	175	95 (88–102)	116	90 (84–84.5)	291	93 (92–95)
< **80 cm**	2	1.1 (^a^)	20	17.2 (11.2–24.9)	22	7.6 (4.9–11)
≥ **80 cm**	173	98.9 (96.4–99.8)	96	82.8 (75.1–88.8)	269	92.4 (89.0–95.1)
**BMI**	175	30.7 (28.1–33.9)	116	28.1 (25.2–30.1)	291	29.7 (26.8.0–32.3)
< **25 kg/m**^**2**^	7	4.0 (1.8–7.7)	27	23.3 (16.3–31.6)	34	11.7 (8.4–15.7)
≥ **25 kg/m**^**2**^	168	96.0 (92.3–98.2)	89	76.7 (68.4–83.7)	257	88.3 (84.3–91.6)
**Blood pressure**						
**SBP**	175	124 (115–139)	116	115 (108–122)	291	120 (111–132)
**DBP**	175	76 (68–83)	116	70.5 (66–75)	291	74 (67–80)
**SBP < 130/DBP < 85 mm Hg**	90	51.4 (44.1–58.8)	108	93.1 (87.4–96.7)	198	68.0 (62.5–73.2)
**SBP ≥ 130/DBP ≥ 85 mm Hg**	85	48.6 (41.2–55.9)	8	6.9 (3.3–12.6)	93	32.0 (26.8–37.5)
**Triglycerides**	175	201 (166–283)	116	123 (96–149)	291	174 (126–228)
< **150 mg/dL**	22	12.6 (8.3–18.1)	90	77.6 (69.4–84.4)	112	38.5 (33.0–44.2)
≥ **150 mg/dL**	153	87.4 (81.9–91.7)	26	22.4 (15.6–30.6)	179	61.5 (55.8–67.0)
**HDL-c**	175	42.5 (37.9–48.1)	116	50.1 (40.4–55.1)	291	44.3 (38.9–51.2)
≥ **50 mg/dL**	26	14.9 (10.2–20.7)	60	51.7 (42.7–60.7)	86	29.6 (24.5–35.0)
< **50 mg/dL**	149	85.1 (79.3–89.8)	56	48.3 (39.3–57.3)	205	70.4 (65.0–75.5)
**Glucose**	175	92 (83.5–104)	116	83 (76–89.5)	291	88.0 (80.5–97.5)
< **100 mg/dL**	118	67.4 (60.2–74.0)	110	94.8 (89.7–97.8)	228	78.4 (73.4–82.8)
≥ **100 mg/dL**	57	32.6 (26.0–39.8)	6	5.2 (2.2–10.3)	63	21.6 (17.2–26.6)

^a^Not available

Among women with MetS, 99% had a waist circumference greater than 80 cm, 96% presented overweight or obesity, 87% had high levels of triglycerides, and 85% had low HDL-c levels. Thirty-three percent presented hyperglycemia and 49% HBP.

### Comorbidities and frequency of food consumption according to MetS

Women with MetS had a higher prevalence of T2D, hypertension, and polycystic ovary syndrome than women without MetS (Table 3). They consumed the following food groups five or more times per week: dairy products (41%), fruits (40%), vegetables (37%), red meat (3.5%), poultry (1.7%), cereals and tubers (100%), legumes (75%), and sugar-sweetened beverages (13.3%). No statistically significant differences between food groups were observed between women with and without MetS.

**Table 3:** Comorbidities and frequency of food intake by metabolic syndrome in women from Chiapas, México

**Variables**	**With MetS (*n*)**	**% (95% CI)**	**Without MetS (*n*)**	**% (95% CI)**	**Total**	**% (95% CI)**
**T2D**	18	10.3 (6.4–15.4)	3	2.6 (^a^)	21	7.2 (4.7–10.6)
**Hypertension**	85	48.6 (41.2–55.9)	8	6.9 (3.3–12.6)	93	32 (26.8–37.5)
**Polycystic ovary syndrome**	20	11.4 (7.4–16.8)	11	9.5 (5.1–15.8)	31	10.7% (7.5–14.6)
**Smoking**	2	1.1 (^a^)	2	1.7 (^a^)	4	1.4 (^a^)
**Alcoholic beverage consumption**	52	29.7 (23.3–36.8)	43	31.7 (28.7–46.1)	95	32.6 (27.5–38.2)
***Frequency of food group intake***						
**Dairy products**						
0–1 times per week	51	29.5 (23.1–36.6)	31	27.2 (19.7–35.9)	82	28.6 (23.6–34.0)
2–4 times per week	51	29.5 (23.1–36.6)	45	39.5 (30.9–48.6)	96	33.4 (28.2–39.1)
≥ 5 times per week	71	41.0 (33.9–48.5)	38	33.3 (25.2–42.3)	109	38.0 (32.5–43.7)
**Fruits**						
0–1 times per week	36	20.8 (15.3–27.3)	26	22.8 (15.8–31.1)	62	21.6 (17.1–26.6)
2–4 times per week	68	39.3 (32.3–46.7)	32	28.1 (20.4–36.8)	100	34.8 (29.5–40.5)
≥ 5 times per week	69	39.9 (32.8–47.3)	56	49.1 (40.1–58.2)	125	43.6 (37.9–49.3)
**Vegetables**						
0–1 times per week	27	15.6 (10.8–21.6)	21	18.4 (12.1–26.3)	48	16.7 (12.8–21.4)
2–4 times per week	82	47.4 (40.1–54.8)	52	45.6 (36.7–54.8)	134	46.7 (41.0–52.5)
≥ 5 times per week	64	37.0 (30.1–44.4)	41	36.0 (27.6–45.0)	105	36.6 (31.2–42.3)
**Red meats**						
0–1 times per week	106	61.3 (53.9–68.3)	65	57.0 (47.8–65.8)	171	59.6 (53.8–65.1)
2–4 times per week	61	35.3 (28.4–42.6)	42	36.8 (28.4–45.9)	103	35.9 (30.5–41.6)
≥ 5 times per week	6	3.5 (1.5–7.0)	7	6.1 (2.8–11.7)	13	4.5 (2.6–7.4)
**Poultry**						
0–1 times per week	94	54.3 (46.9–61.6)	60	52.6 (43.5–61.6)	154	53.7 (47.9–59.4)
2–4 times per week	76	43.9 (36.7–51.4)	48	42.1 (33.3–51.3)	124	43.2 (37.6–49.0)
≥ 5 times per week	3	1.7 (^a^)	6	5.3 (2.2–10.5)	9	3.1 (1.6–5.6)
**Fish and shellfish**						
0–1 times per week	153	88.4 (83.0–92.6)	104	91.2 (85.0–95.4)	257	89.5 (85.6–92.7)
2–4 times per week	20	11.6 (7.4–17.0)	9	7.9 (4.0–13.9)	29	10.1 (7.0–14.0)
≥ 5 times per week	0	0 (^a^)	1	0.9 (^a^)	1	0.3 (^a^)
**Cereals and tubers**^**a**^						
0–1 times per week	0	0 (^a^)	1	0.9 (^a^)	1	0.3 (^a^)
≥ 5 times per week	173	100	113	99.1 (96.0–99.9)	286	99.7 (98.4–100.0)
**Legumes**						
0–1 times per week	8	4.6 (2.2–8.5)	10	8.8 (4.6–15.0)	18	6.3 (3.9–9.5)
2–4 times per week	35	20.2 (14.8–26.7)	31	27.2 (19.7–35.9)	66	23.0 (18.4–28.1)
≥ 5 times per week	130	75.1 (68.3–81.1)	73	64.0 (55.0–72.4)	203	70.7 (65.3–75.8)
**Sugar-sweetened beverages**						
0–1 times per week	95	54.9 (47.5–62.2)	64	56.1 (47.0–65.0)	159	55.4 (49.6–61.1)
2–4 times per week	55	31.8 (25.2–39.0)	36	31.6 (23.6–40.5)	91	31.7 (26.5–37.3)
≥ 5 times per week	23	13.3 (8.9–18.9)	14	12.3 (7.2–19.2)	37	12.9 (9.4–17.1)

^a^Not available

### Allele and genotype frequencies of the study population

Table 4 shows the allele frequencies of the rs9939609-FTO analyzed in our study population and their comparison with those reported in other studies for main blocks of the population (American, European, East Asian, African) and Mexican population. In our study, the SNP rs9939609 was in HWE (*p* > 0.05). TT genotype was identified in 233 samples (80%), TA in 54 samples (18%), and AA in 4 samples (1%). The frequency of the A allele was 0.10.

**Table 4:** Comparison of the allele frequencies of rs-9939609/FTO between our study population with other population studies

**Gene/SNP**		**Allele frequency in our study**	**Reference Allele Frequency (gnomAD** [22] **and The Page Study** [23]**)**				
	Allele	Women from Chiapas, Mexico	American population^a^	European population^a^	East Asian population^a^	African population^a^	Mexican population^b^
**FTO/RS9939609**	**T**	0.8935	0.6820	0.5924	0.8621	0.5217	0.7442
	*p*-value*		< 0.001	< 0.001	0.1341	< 0.001	< 0.001
	**A**	0.1065	0.3179	0.4075	0.1379	0.4782	0.2558
	*p*-value*		< 0.001	< 0.001	0.1341	< 0.001	< 0.001

^*^Two-sample tests on the equality of proportions

^a^Data obtained from gnomAD

^b^Data obtained from The Page Study

There are statistically significant differences between the frequencies reported in our study and those reported for American, European, African, and Mexican populations. No significant differences were found between the allele frequency reported for the East Asian population and our study population.

### Association between MetS components and different rs9939609-FTO genotypes in the study population

We analyzed different modes of inheritance (co-dominant, dominant, and recessive) for the *rs9939609-FTO* genotypes with regard to MetS components. Table 5 shows the association between rs9939609-FTO and hyperglycemia in the study population. For this analysis, only individuals who presented fasting glucose levels ≥ 100 mg/dL were considered (treatment for elevated glucose was not included as an outcome). A significant association was observed between the rs9939609/FTO and hyperglycemia in the dominant model. The TA/AA genotype carriers were twice more likely to develop hyperglycemia than those with the TT genotype (OR = 2.6; 95% CI 1.3–5.3, *p* = 0.007).

**Table 5:** Associations between rs9939609-*FTO* and hyperglycemia in women from Mayan communities of Chiapas, Mexico

**SNP/gene**	**Genotypes *n* (%)**			**Dominant model**^**a**^	
***rs9939609-FTO***	TT	TA	AA	OR (95% CI)	*p*-value
***Normal glycemia* (serum glucose < 100 mg/dL)**	189 (64.9)	35 (12.0)	4 (1.4)	**2.6** (1.3–5.3)	0.007
***Hyperglycemia* (serum glucose ≥ 100 mg/dL)**	44 (15.1)	19 (6.5)	0 (0)		

^a^The model was adjusted for age (years, continuous), BMI (kg/m^2^, continuous), schooling (years, continuous), and presence of T2D

No significant interactions were observed for the geographic region or previously diagnosed hypertension (*p* for interaction > 0.05).

## Discussion

MetS has been associated with an increased risk of developing CVD and T2D [21]. In Mexico, some studies have been conducted to estimate the proportion of the Mexican population with MetS. For instance, Aguilar-Salinas et al. [24] reported in 2004 a prevalence of 14% according to the World Health Organization (WHO) criteria and 27% according to the National Cholesterol Education Program Adult Treatment Panel III (NCEP-ATPIII) criteria in population from 20 to 69 years of age. González-Villalpando et al. [14] in a study performed among the Mexican diabetic population reported MetS prevalence of 39.9% and 59.9% in males and females, respectively, based on the NCEP-ATPIII criteria. It is noteworthy that previous studies were carried out in other Mexican states such as Guanajuato, Jalisco, Puebla, Baja California Norte, Morelos, Querétaro, and Mexico City [25]. By contrast, in the southeast region of the country with a high proportion of the indigenous population, only few studies have been carried out on this topic. As an example, the study conducted by Castro et al. in 2011 identified a prevalence of 49% of MetS in adults from Merida, Yucatan, according to the IDF criteria [26]. We found that 60% of the participants in our study presented MetS, under the criteria published by Alberti et al. in 2009 [21], which represents a much higher percentage than the previously mentioned for the Mayan population [26], and it is even higher than the one reported for the overall Mexican population [27]. Our study population is conformed by a high percentage of native peoples (35%), with low educational levels (36%). Moreover, they live in poor household conditions with low availability of public services. They belong to population groups who in the last decades have changed their diet and physical activity, adopting habits and activities that predispose them to suffer various diseases [28], especially those of cardiovascular and endocrine-metabolic nature. As mentioned above, the study population is a vulnerable population at high risk to develop these important diseases, due to the interaction of several risk factors such as diet, physical activity, and socioeconomic level, among others.

Regarding the frequency of the variant rs9939609 of the FTO gene, we found differences between our results with those previously reported for American, European, African [23], and Mexican populations [22, 29]. This could be due to the distinction among ethnic groups analyzed. Nevertheless, it cannot be ruled out that different sample sizes might explain such differences in the results. In the case of allele frequencies reported for the East Asian population [23], no differences were observed when compared with our results.

This FTO variant has been extensively studied because it presents a strong association with obesity markers, i.e., a 3-kg increase of additional body weight for each copy of the risk allele in carriers has been documented in several populations [7]. Additionally, several research lines have linked this SNP in FTO to variations in food consumption patterns [30, 31]. Epidemiological studies suggest a positive association between the risk genotype of FTO and high energy consumption [32, 33], low satiety power [30], higher protein intake [34], and greater preference for high-fat meals [35]. Although further evidence shows contradictory results, for instance, some studies have found less robust associations and outcomes in opposite directions [36–38]. A study among the German population found that SNPs of the FTO were strongly associated with obesity and T2D [9, 39]. Other studies have also demonstrated that the carriers of the A allele were more likely to develop hyperglycemia than their counterparts with the T allele [40–42].

Studies carried out in other regions of Mexico have analyzed the association of FTO polymorphisms with MetS components [17, 43–45]. However, none of them has found a significant association of this polymorphism with hyperglycemia. In our study, we found a statistically significant association between the rs9939609/FTO and hyperglycemia. Women with TA/AA genotypes showed a higher probability of hyperglycemia than women with the TT genotype (*p* = 0.007).

Differences between our results and those reported in the literature may be due to different factors, for example, the ethnicity of the population evaluated, differences in body composition, diet, and the presence of other comorbidities [30].

It is remarkable that no previous studies have been carried out in this Mayan region of Mexico on the relationship of this rs9939609-FTO variant with MetS components. This paper would be the first one to report for Mayan indigenous populations an association between the presence of A allele of rs9939609/FTO and hyperglycemia.

## Conclusions

In our study, the TA/AA genotypes of the rs9939609-FTO polymorphism increased the risk of hyperglycemia among women from three Mayan regions of Chiapas, Mexico. This finding has never been reported before in the Mayan indigenous population from Chiapas, specifically, in women with a high prevalence of MetS (60%).

Thus, this investigation sets up the basis to understand the influence of a common variant on cardiometabolic risk factors among this population.

However, further studies in the Mexican indigenous population are required, particularly in the most vulnerable groups to generate more evidence about this topic.

Finally, on the basis of our results, we recommend to implement effective public health policies to control and prevent the increasing MetS prevalence and its cardiovascular effects among the indigenous population of Mexico.

## Data Availability

The datasets used and/or analyzed during the current study are available from the corresponding author on reasonable request.

## Abbreviations

MetS: Metabolic syndrome
SNPs: Single nucleotide polymorphisms
FTO: Fat mass and obesity-associated gene
IR: Insulin resistance
T2D: Type 2 diabetes
HBP: High blood pressure
BMI: Body mass index
OB: Obesity
HDL-c: High-density lipoprotein cholesterol
SBP: Systolic blood pressure
DBP: Diastolic blood pressure
HWE: Hardy–Weinberg equilibrium
OR: Odds ratio
CI: Confidence intervals
CVD: Cardiovascular disease
WHO: World Health Organization
NCEP-ATPIII: National Cholesterol Education Program Adult Treatment Panel III
